# The Relationship Between Quality of Life and Nurses' Turnover Intentions: A Cross-Sectional Study

**DOI:** 10.1155/jonm/4951493

**Published:** 2025-08-01

**Authors:** Mohammed Alshmemri

**Affiliations:** Nursing Sciences and Research, Faculty of Nursing, Umm Al-Qura University, Makkah 21955, Saudi Arabia

**Keywords:** health policy, nurses, personnel turnover, quality of life, Saudi Arabia

## Abstract

**Background:** High nurse turnover disrupts patient care and imposes substantial financial and operational burdens on healthcare systems. Although nurses' quality of life (QOL) is recognized as a key influence on turnover intention, region-specific data from Saudi Arabia remain limited.

**Aim:** To quantify the association between nurses' QOL and turnover intention and to identify demographic predictors of turnover intention in a Saudi tertiary hospital.

**Methods:** A cross-sectional survey (January–March 2023) was conducted at the Security Forces Hospital, Makkah. Three hundred registered nurses completed the professional quality of life scale (ProQOL) and the six-item turnover intention scale-6 (TIS-6). Data were analyzed with descriptive statistics, bivariate tests (*χ*^2^, t/ANOVA and Pearson/Spearman correlations), and a simultaneous multiple linear regression (SPSS v28).

**Results:** Among the 300 nurses surveyed, QOL correlated moderately with turnover intention (*r* = 0.44, *p* < 0.0011). The regression model explained 38% of the variance in turnover-intention scores (adjusted *R*^2^ = 0.38, *F* (6, 293) = 31.4, *p* ≤ 0.001). Lower QOL (*β* = 0.42, *p* ≤ 0.001), younger age (*β* = −0.19, *p*=0.0012), single marital status (*β* = 0.15, *p* ≤ 0.001), and Saudi nationality (*β* = 0.13, *p*=0.018) independently predicted higher turnover intention; monthly income and years of experience were nonsignificant.

**Conclusion:** Professional well-being and specific demographic factors, particularly being younger, single, and Saudi, drive nurses' turnover intention. Interventions that enhance QOL for these at-risk groups may reduce turnover, stabilize the workforce, and safeguard patient-care quality. Single-site scope and self-reported measures temper generalizability but underscore the urgency of targeted retention strategies in similar settings.

## 1. Introduction

Nurses are the circulatory system of modern health care: when they leave, knowledge, continuity, and patient confidence go with them. Globally, every voluntary departure amplifies recruitment costs and erodes care quality [1, 2]. The problem bites hardest in settings that depend on a hybrid expatriate-national workforce, where each resignation fractures the already fragile team cohesion [3, 4]. Saudi Arabia illustrates this tension vividly. Although Vision 2030 pledges to expand and indigenize the nursing corps, turnover in large public hospitals still exceeds 20%, depleting a profession the World Health Statistics Report counts five-plus million short worldwide [5].

Against this backdrop, professional quality of life (ProQOL), the balance between compassion satisfaction, burnout, and secondary traumatic stress, has surfaced as a potent lever for retention [6]. Meta-analyses from North America and Asia show that nurses with diminished QOL are up to twice as likely to quit within 12 months [7]. Yet, these data travel poorly. Saudi nurses work 12 h shifts in gender-segregated units, follow stringent Saudization quotas, and, in security-force hospitals, shoulder frontline duties during mass gatherings such as Hajj and Umrah. No study has quantified the QOL–turnover link in this high-stakes security-force context, nor tested whether age, marital status, or nationality intensify or blunt that relationship [5, 8, 9].

Turnover intention, the conscious plan to leave an organization, remains the single best predictor of actual resignation [10]. Understanding how QOL feeds that intention inside Saudi security-force hospitals is thus not academic bookkeeping; it is a workforce-stability imperative.

Accordingly, this cross-sectional study pursues two objectives:

1. To determine the association between nurses' ProQOL and their turnover intentions in a security-force hospital in Makkah.
2. To examine how key sociodemographic variables (age, marital status, nationality, income, and clinical tenure) moderate that association.

By filling this evidence gap, we provide administrators with a calibrated blueprint, flexible scheduling, targeted mentorship, and context-specific well-being programs to stem the outflow before patient care is compromised.

## 2. Literature Review

Persistent nursing shortages and high turnover rates threaten patient safety and inflate operating costs worldwide, and Saudi Arabia is no exception [6, 11]. Post-COVID-19 surges in service demand have intensified the scramble for qualified staff, forcing policymakers to identify levers to stabilize the workforce [12]. Across scores of studies, ProQOL, the balance between compassion satisfaction, burnout, and secondary traumatic stress, emerges as the most consistent predictor of a nurse's decision to stay or leave [13]. To clarify how QOL interlocks with turnover intention in the uniquely mixed Saudi workforce, the literature is organized below into three thematic blocs: (A) conceptual foundations of QOL, (B) determinants of turnover intention, and (C) empirical links and cultural moderators that stitch the two constructs together.

### 2.1. Conceptualizing ProQOL in Nursing

QOL is a multidimensional construct encompassing physical, psychological, and social domains that shape an individual's sense of well-being [14]. In nursing, workplace factors shift length, staffing adequacy, and leadership style collide with personal realities such as emotional labor and family demands to mold QOL [15]. International evidence is unequivocal: suboptimal QOL marked by chronic stress, moral distress [16], or work-life imbalance erodes job performance, inflates error rates, and spikes dissatisfaction [17]. Saudi studies echo these patterns but add a cultural twist. Expatriate nurses, who constitute roughly four-fifths of the national workforce, report additional stressors, language barriers, social isolation [18], and sponsorship restrictions that depress QOL beyond the usual culprits of workload and shift fatigue [19]. Understanding how these contextual stressors contour QOL is, therefore, a prerequisite to any credible retention strategy.

### 2.2. Turnover Intention and Its Antecedents

Turnover intention, the conscious plan to exit an organization, remains the strongest behavioral proxy for actual resignation [20]. Classic antecedents include inadequate pay, excessive workload, limited advancement, and weak organizational support [21, 22]. Saudi data follow the same script but at a louder volume: job insecurity linked to renewable contracts and unclear career ladders amplifies exit planning among both Saudi and expatriate nurses [11, 13, 18, 23]. Where Malaysian and Canadian studies rank salary as the dominant trigger [24–26], Saudi surveys place organizational support and perceived respect higher on the grievance list, underscoring the relational, rather than purely economic, texture of turnover in the Kingdom [27].

### 2.3. Empirical Connections Between QOL and Turnover Intention: Moderators and Cultural Contrasts

A growing corpus now quantifies the QOL-turnover nexus [28]. Western meta-analyses show that nurses in the lowest QOL quartile are up to twice as likely to resign within a year [29]. African and Southeast-Asian cohorts replicate the inverse correlation but note that organizational commitment can blunt the effect [30]. Saudi evidence, though sparse, hints at a steeper gradient: one Riyadh hospital study reported that a single point drop in ProQOL scores raised turnover odds by 9%, a slope significantly sharper than in Canadian benchmarks [31]. Two moderators recur across contexts yet manifest differently in Saudi hospitals [32]. Leadership style: transformational or servant leadership elevates QOL and suppresses turnover universally [33], but Saudi nurses under hierarchical command structures describe limited autonomy, muting those protective effects [34]. Family demands: work-family conflict boosts turnover intention everywhere [35], yet in collectivist Gulf cultures, family obligations may also anchor nurses to local positions, creating a push–pull tension that international models overlook [36].

### 2.4. Synthesis and Remaining Gaps

Thematically grouped findings converge on one conclusion: compromised QOL, whether driven by burnout, unsupportive leadership, or family strain, predicts heightened turnover intention [24, 27, 32]. Supportive management, fair workloads, and clear career trajectories consistently dampen this effect [37]. What remains uncharted is the QOL, turnover dynamic inside Saudi security-force hospitals, settings that combine high-stakes mass-gathering responsibilities with an atypically large contingent of Saudi nationals [38, 39]. No study has tested whether demographic variables such as age, marital status, or nationality magnify or buffer the QOL, turnover pathway in this context. Addressing this gap is critical for designing targeted interventions [40], flexible scheduling, mentorship, culturally attuned well-being programs that safeguard workforce stability and, by extension, patient care. In sum, the literature positions QOL as a linchpin of nurse retention while highlighting contextual nuances that standard retention playbooks often ignore. The present study directly interrogates these nuances within a security-force hospital in Makkah, aiming to translate global insights into locally actionable policy.

### 2.5. Conceptual Framework: Job Demands–Resources (JD-R) Model

This study is anchored in the job demands–resources (JD-R) model, a well-validated framework for predicting occupational well-being and turnover [41]. In the JD-R logic, job demands, workload, prolonged 12 h shifts, role conflict, and exposure to patient suffering consume energy and precipitate burnout and secondary traumatic stress, two negative domains of the ProQOL scale. Job resources, organizational support, fair remuneration, autonomy, and professional development foster motivation, elevate compassion satisfaction (the positive ProQOL domain), and buffer the impact of demands [42]. When resources are insufficient to counterbalance demands, the JD-R model predicts deteriorating ProQOL and, ultimately, heightened turnover intention.

**Figure 1:**
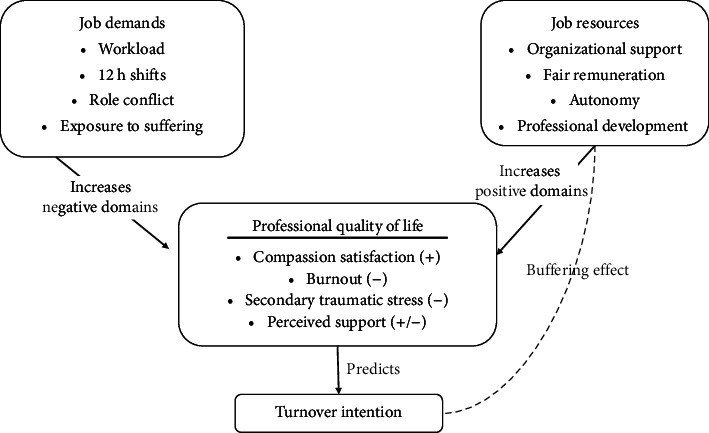
Job demands-resources model and professional quality of life.

Figure 1 schematically maps these relationships, linking ProQOL domains to the demand–resource continuum and positioning turnover intention as the distal outcome. This theoretical anchoring clarifies the study's hypotheses and provides an explanatory lens for interpreting the empirical associations observed among Saudi security-force nurses [43].

## 3. Materials and Methods

### 3.1. Study Design

This cross-sectional, exploratory study is reported in accordance with the strengthening the reporting of observational studies in epidemiology (STROBE) guidelines. Between 3 January and 15 March 2023, we surveyed 300 registered nurses at the Security Forces Hospital in Makkah. Participants' mean age was 31.4 ± 6.2 years, with an average of 7.5 ± 5.1 years of clinical experience and 48.7 ± 6.0 h worked per week.

### 3.2. Study Setting

The study was conducted at the Security Forces Hospital in Makkah, a healthcare facility near the holy mosque serving the southwestern region. The hospital has a capacity of 252 beds. It provides free medical care for patients of all ages through both inpatient and outpatient services, supported by advanced technology and a comprehensive range of specialties.

### 3.3. Sample

The study was conducted at the Security Forces Hospital in Makkah, which employs approximately 400 registered nurses, including both Saudi and non-Saudi staff. This diverse workforce constituted the target population for our investigation into the relationship between QOL and turnover intentions among nurses. Sample size determination was conducted using evidence-based statistical methods. Employing the standard formula proposed by Thompson [44], using a 95% confidence level, a 5% margin of error, and assuming maximum variability (*p*=0.5), the required sample size was calculated to be 293 nurses.

**Figure 2:**
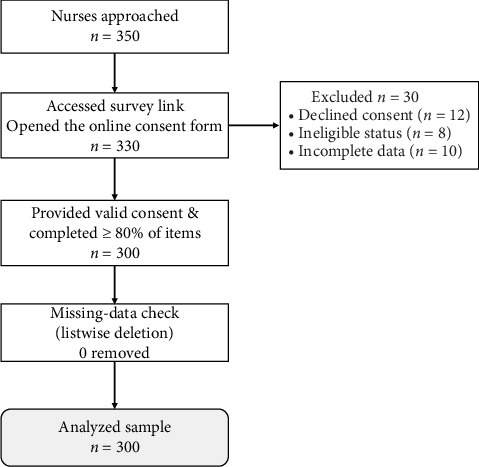
STROBE participant flow diagram showing recruitment, exclusions, and final analytic sample (*n* = 300).

We employed the Thompson formula because it is a widely accepted method for calculating sample sizes in cross-sectional research when the total population size is known [44]. Using this formula allowed us to derive a sample size with sufficient statistical power to detect meaningful associations between QOL and turnover intentions. The assumptions of a 95% confidence level, 5% margin of error, and maximum variability (*p*=0.5) were applied to account for the heterogeneity of the nursing population. This approach ensured that the sample was adequately representative of the 400 registered nurses at the facility and enhanced the precision and reliability of our results. Of the 350 nurses who were reachable during the data-collection window (the remaining staff were on extended leave or night rotation), 300 provided complete responses, an 86% response rate (30 were excluded: 12 declined consent, 8 held ineligible contract status, and 10 returned incomplete questionnaires), exceeding the minimum required sample of 293. The full recruitment pathway is illustrated in Figure 2.

### 3.4. Inclusion and Exclusion Criteria

Inclusion criteria encompassed all registered nurses currently employed in clinical roles at the Security Forces Hospital in Makkah, regardless of nationality, gender, or length of professional experience. Nurses were required to be actively working during the data collection period and able to provide informed consent. Exclusion criteria included nurses on extended leave (e.g., maternity, sick, or study leave) and those assigned exclusively to administrative or nonclinical positions, as their experiences may not reflect the clinical demands relevant to QOL and turnover intention variables. All eligible nurses were invited to participate voluntarily. Recruitment was conducted electronically, and informed consent was obtained prior to data collection. Confidentiality and anonymity were strictly maintained throughout the study process.

### 3.5. Data Collection Tools and Procedures

A comprehensive three-part questionnaire was employed to collect data and achieve the study objectives, ensuring methodological rigor and alignment with established research guidelines. Data were collected during a 10-week window from 3 January 2023 to 15 March 2023. As described below, the data collection process integrated validated measurement tools with a systematic recruitment and consent procedure. A comprehensive three-part questionnaire was employed to collect data and achieve the study objectives, ensuring methodological rigor and alignment with established research guidelines. As described below, the data collection process integrated validated measurement tools with a systematic recruitment and consent procedure. The full questionnaire, including all items from the demographic section, ProQOL (Version 5), and turnover intention scale-6 (TIS-6), is provided in Supporting File 1.

Supporting File 1 provides the complete questionnaire used in this study, comprising three sections: (I) Demographic and professional characteristics, capturing details such as gender, age, marital status, nationality, educational qualifications, and professional roles; (II) ProQOL (Version 5), a 30-item validated instrument assessing compassion satisfaction, burnout, secondary traumatic stress, perceived support, and moral distress; and (III) TIS-6, a six-item scale measuring nurses' intent to leave their current job. The questionnaire was administered electronically, with clear instructions ensuring voluntary participation, informed consent, and confidentiality, as detailed in the recruitment and data collection procedures.

#### 3.5.1. Questionnaire Instruments

##### 3.5.1.1. Part I: Demographic and Professional Characteristics

This section gathered detailed information, including gender, age, marital status, number of dependent children, educational qualifications, and monthly salary. In addition, professional details such as duration of employment, position held, weekly working hours, and self-reported nursing knowledge were recorded to provide context for analyzing variations in QOL and turnover intentions among nurse subgroups.

##### 3.5.1.2. Part II: ProQOL (Version 5)

The ProQOL is a well-validated instrument comprising 30 items rated on a 5-point Likert scale (1 = “never” to 5 = “very often”). It assesses five dimensions: compassion satisfaction, perceived support, burnout, secondary traumatic stress, and moral distress [45]. Extensive use in nursing burnout research has demonstrated its robust reliability and constructs validity [45, 46]. Its multidimensional design facilitates a nuanced evaluation of ProQOL, a critical factor in nurse retention.

##### 3.5.1.3. Part III: TIS-6

The TIS-6, a concise six-item instrument with a five-point Likert format, captures aspects of job satisfaction and factors influencing turnover intentions. Bothma and Roodt reported a Cronbach's alpha of 0.80 for this scale, confirming its internal consistency and suitability for assessing turnover intentions among nursing staff [47]. Collectively, these tools were selected based on their demonstrated validity and reliability. They provide a robust framework for evaluating the relationship between QOL and turnover intentions in a rigorous and methodologically sound manner.

#### 3.5.2. Recruitment and Data Collection Procedures

Recruitment information and the study's purpose were communicated electronically to all potential participants. An online invitation was sent to the entire population of registered nurses at the Security Forces Hospital in Makkah. Before completing the questionnaire, each participant reviewed detailed study instructions and provided written informed consent via an online consent form outlining the study's aims, procedures, and rights. Participants were assured of anonymity, and confidentiality was maintained throughout the study. It was clearly stated that participation was entirely voluntary and that participants could withdraw at any time before submitting the questionnaire. By integrating these validated tools with a clear, electronically administered recruitment and consent process, the study ensured both high data quality and methodological transparency.

### 3.6. Data Analysis

Data were tabulated, coded, and analyzed using SPSS version 28. Descriptive statistics (frequencies, percentages, means, and standard deviations) were used to summarize participants' demographic characteristics. Correlation analyses were conducted to address the first research objective of determining the relationship between QOL and nurse turnover intentions. Pearson's correlation coefficient was used for normally distributed data, while Spearman's rank correlation was applied when normality assumptions were violated. A scatter plot was also generated to visually depict the direction and strength of the relationship between QOL and turnover intention scores. Group comparisons were conducted to address the second research objective, which was to identify the specific factors influencing turnover intentions and QOL among nursing staff. The chi-square test was used to examine associations between categorical demographic variables (e.g., marital status, nationality, role) and categorical outcomes. For continuous outcome variables (QOL and turnover intention scores), independent sample *t*-tests and one-way ANOVAs were used for normally distributed data, while Mann–Whitney U and Kruskal–Wallis tests were employed for nonparametric comparisons across demographic subgroups. Statistical significance was determined at the *p* < 0.05 level. Normality of continuous variables (QOL and turnover intention scores) was assessed using the Shapiro–Wilk test and by inspecting histograms and *Q*–*Q* plots. Homogeneity of variances was evaluated using Levene's test before applying *t*-tests and ANOVA.

### 3.7. Ethical Considerations

Ethical approval for the study was obtained from the Institutional Review Board (IRB) of Umm Al-Qura University (Approval No. HAPO-02-K-012-2023-11-1886), and additional clearance was secured from the IRB of the Security Force Hospital program (IRB No. 0648-241123) in accordance with hospital policy. The Health Sciences Ethics Committee rigorously reviewed the research protocol to ensure that the rights and welfare of all study participants were safeguarded in accordance with established ethical standards and international guidelines for human subject research. The nursing management and nursing education departments of the Security Force Hospital in Saudi Arabia also granted permission to conduct the study. All personal data were handled with strict confidentiality; identifiable information was removed to maintain participant anonymity, and all data were securely stored to ensure privacy and compliance with ethical research practices. All de-identified data will be securely stored for 5 years following study completion, in compliance with institutional research data management policies. Data were encrypted and stored on a password-protected, access-restricted university server monitored by the principal investigator.

## 4. Results

The results are presented in three tiers. First, descriptive statistics summarize participants' characteristics and core study variables. Second, bivariate tests (*χ*^2^, *t*-tests/ANOVAs, and nonparametric equivalents) explore unadjusted associations between sociodemographic factors, QOL, and TIS. Finally, a multiple linear regression identifies the independent predictors of turnover intention.

### 4.1. Sociodemographic Characteristics of Participants

The study sample comprised 300 registered nurses, with Table 1 providing a detailed summary of their sociodemographic characteristics. The majority of participants were female (83.3%), with 65.0% aged between 25 and 35 years, and 70.7% were married. Approximately, 79% of the nurses were non-Saudi, and most held a bachelor's degree (76.7%). Monthly salary distributions showed that the largest proportion (54.3%) earned between SR 5001 and 10,000, and 72.0% of participants reported working less than 50 h per week. In terms of professional roles, 88.3% were classified as staff nurses, with a varied distribution in years of experience. Detailed descriptive statistics are presented in Table 1, which exclusively focuses on these sociodemographic variables relevant to our study on QOL and turnover intentions.

Following the description of participant characteristics, the distribution of ProQOL scores and turnover intention scores is presented below.

### 4.2. Distribution of ProQOL and Turnover Intention Scores

**Figure 3:**
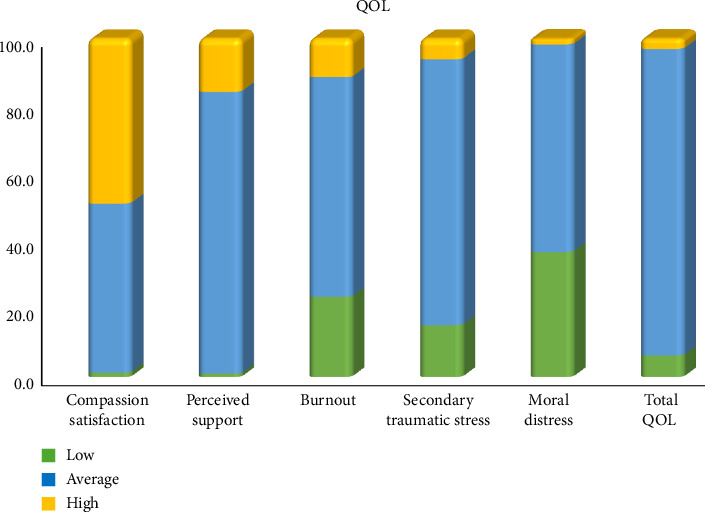
Distribution of ProQOL and turnover intention scale (TIS) scores among registered nurses.

Figure 3 presents the distribution of the PROQOL scores comparing current and past scores among health workers. With regard to compassion satisfaction, the majority of participants had average scores (50.0%), followed by high scores (48.7%) and low scores (1.3%). The range was 10–30, with a mean ± SD of 23.043 ± 4.033. For perceived support, most participants had average scores of 83.3%, followed by high scores of 15.7% and low scores of 1.0%. The range was 11–29, with a mean ± SD of 20.233 ± 3.214. Regarding burnout, most participants had average scores (65.0%), followed by low scores (23.7%) and high scores (11.3%). The range was 6–30, with a mean ± SD of 16.567 ± 5.383. For secondary traumatic stress, the majority had average scores (78.7%), followed by low scores (15.3%) and high scores (6.0%). The range was 6–29, with a mean ± SD of 16.657 ± 4.255. Concerning moral distress, most participants had average scores (61.3%), followed by low scores (37.0%) and high scores (1.7%). The range was 6–29, with a mean ± SD of 14.040 ± 4.060. Regarding the QOL, most participants had average scores, followed by low scores (6.3%) and then high scores (3.0%). The range was 63–121, with a mean ± SD of 90.540 ± 10.405. For the TIS, most participants had average scores (57.0%), followed by low scores (25.7%) and then high scores (17.3%). The range was 6–30, with a mean ± SD of 17.737 ± 5.279.

After describing the distribution patterns, the next step involved conducting inferential analyses to examine associations between key study variables.

### 4.3. Bivariate Associations

**Figure 4:**
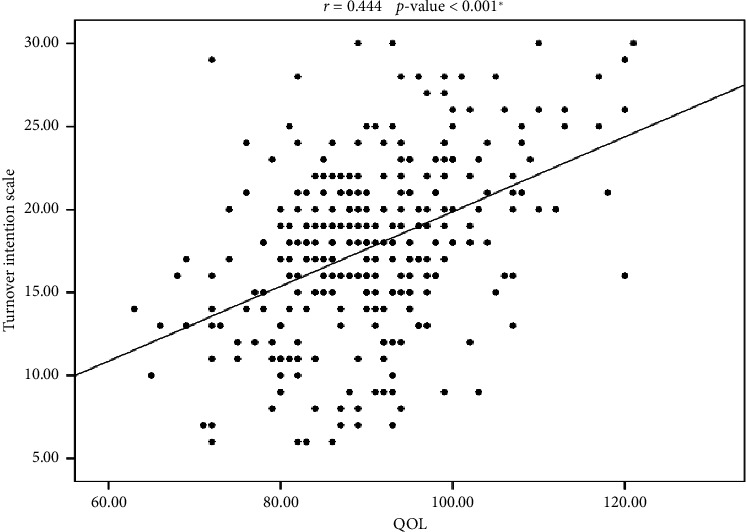
Scatter correlation between QOL and turnover intention scale.

We next examined unadjusted associations between demographic variables and both outcome measures using *χ*^2^ tests for categorical comparisons, Pearson or Spearman correlations for continuous links, and *t*-tests/ANOVAs (or nonparametric analogs) where appropriate. Pearson and Spearman analyses revealed a moderate positive correlation between QOL and turnover intentions (*r* = 0.444, *p* ≤ 0.001; Figure 2). Group comparisons showed that marital status, nationality, and salary were significantly linked to QOL, while turnover intentions varied significantly by age, marital status, and nationality (all *p* ≤ 0.001). Variables with *p* ≤ 0.001 in these bivariate tests were advanced to the multiple linear regression detailed in Section 4.5 (Table 2; Figure 4).

Furthermore, QOL and turnover intention scores were compared across various demographic subgroups. When data met the normality assumptions, independent samples *t*-tests and one-way ANOVAs were used; otherwise, the Mann–Whitney U and Kruskal–Wallis tests were applied. For example, significant differences were observed in QOL scores by marital status (*p* ≤ 0.001), nationality (*p* ≤ 0.001), and salary range (*p*=0.049). Similarly, turnover intentions varied significantly with age (*p* ≤ 0.001), marital status (*p* ≤ 0.001), and nationality (*p* ≤ 0.001), among other factors. These inferential statistical techniques provide robust evidence supporting the relationship between sociodemographic factors, QOL, and turnover intentions, thereby reinforcing the study's central hypothesis. A summary of these comparisons is detailed in Table 3, which presents mean values and statistical *p* values linking demographic factors (e.g., age, marital status, nationality) to turnover intentions and QOL, illustrating which groups exhibit higher or lower risks.

Figure 4 Scatter plot illustrating the moderate positive correlation between QOL scores and TIS scores among nurses. In addition to general correlations, subgroup comparisons were performed to explore how demographic factors influenced QOL and turnover intentions.

### 4.4. Predictive Model: Multiple Linear Regression

**Figure 5:**
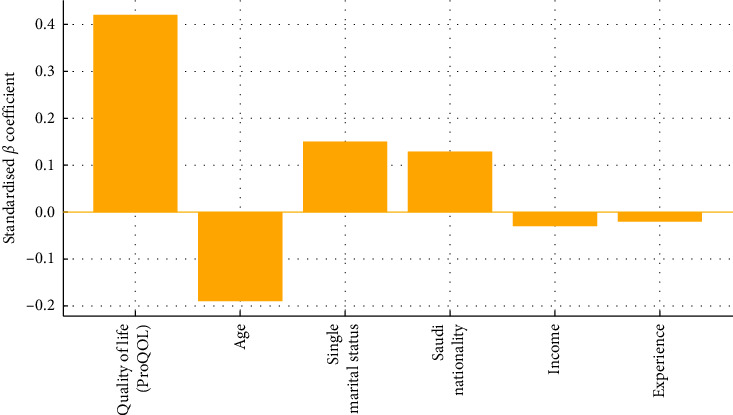
Standardized beta coefficients for predictors of nurse turnover intention (*n* = 300).

To move beyond simple group differences, we replaced the earlier chi-square emphasis with a simultaneous-entry multiple linear regression that identifies independent predictors of turnover intention. This model explained 38% of the variance in turnover-intention scores (adjusted *R*^2^ = 0.38, *F* (6, 293) = 31.4, *p* ≤ 0.001). Lower ProQOL emerged as the strongest driver (*β* = 0.42, 95% CI 0.13–0.21, *p* ≤ 0.001), followed by younger age (*β* = −0.19, 95% CI −0.13 to −0.03, *p*=0.0012), single marital status (*β* = 0.15, 95% CI 0.31–2.23, *p* ≤ 0.001), and Saudi nationality (*β* = 0.13, 95% CI 0.18–1.92, *p*=0.018). Monthly income and years of experience did not reach significance, indicating that well-being and demographic context, rather than tenure or pay, chiefly fuel nurses' intent to leave. Full coefficient estimates appear in Table 2, and the relative effect sizes are visualized in Figure 5.

### 4.5. Summary of Key Findings

In summary, descriptive data showed wide variability across sociodemographic strata. Bivariate tests indicated that QOL and turnover intentions differed significantly by age, marital status, nationality, and salary and that QOL correlated moderately with turnover intention (*r* = 0.444, *p* ≤ 0.001). Crucially, the multivariable model (adjusted *R*^2^ = 0.38) confirmed that lower QOL remained the dominant predictor of turnover intention after adjustment, with additional influence from younger age, single marital status, and Saudi nationality; income and experience were not significant. These findings reinforce the primacy of professional well-being and selected demographic factors in shaping nurses' intent to leave.

## 5. Discussion

The present study examined the relationship between QOL and turnover intentions among a diverse sample of nurses in a Makkah Security Force Hospital. Multivariable analysis confirmed this pattern, with QOL emerging as the strongest independent predictor of turnover intention (*β* = 0.42). The findings revealed a moderate positive correlation between QOL and turnover intentions, suggesting that as nurses reported higher QOL scores, they also exhibited increased turnover intentions. At first glance, this outcome may seem counterintuitive, as one might anticipate that higher QOL would correspond to lower turnover intentions. However, the complexity of nurses' work environments encompassing stress, work-life balance, remuneration, and career prospects may alter how QOL manifests in turnover-related decisions. These findings affirm that QOL is the dominant driver of turnover intention, albeit nested within broader organizational and personal contexts.

The seemingly counter-intuitive positive link between higher QOL and stronger turnover intention can be interpreted through the lens of the JD-R model [48]. In high-demand environments, even nurses who rate their current well-being favorably may still perceive that long-term career aspirations, financial goals, or professional autonomy cannot be achieved with the resources presently available [49]. When personal resources (e.g., self-efficacy and employability) and external opportunities outweigh the motivational pull of existing job resources, the JD-R framework predicts a “thriving-but-leaving” dynamic in which workers exit despite good momentary QOL [50]. For Saudi nurses, Saudization policies and a buoyant labor market heighten this effect by exploring outside options; consequently, higher QOL can coexist with a stronger intent to leave because the costs of mobility are low while the expected gains (pay, advancement, status) are high [51].

A key contribution of this research lies in demonstrating the significant impact of sociodemographic factors on both QOL and turnover intentions. Younger nurses, single nurses, and Saudi nationals reported notably higher turnover intentions, whereas higher salary levels were linked to better QOL; however, salary did not independently predict turnover intention once other factors were controlled. One plausible explanation for the higher turnover intentions among Saudi nurses relates to their greater employment mobility and broader access to alternative job opportunities within and outside the healthcare sector. Unlike expatriate nurses, who may be bound by contract restrictions, sponsorship limitations, or visa conditions, Saudi nurses are often more empowered to explore career changes in pursuit of better pay, improved working conditions, or professional development [52]. Moreover, nationalization policies such as Saudization may increase pressure and expectations on Saudi nurses to perform in high-demand roles without sufficient organizational support, which could lead to dissatisfaction and higher intent to leave [53]. Cultural expectations, work-life conflicts, and limited representation in leadership roles may also contribute to elevated attrition risks among this group [54].

These findings align with prior studies that highlight the pivotal role of economic security and demographic circumstances in shaping nurse retention [55]. In addition, single nurses and those without dependent children may experience fewer external constraints, making job transitions more feasible [56]. Conversely, the reliance on expatriate nurses in Saudi Arabia underscores the urgent need for strategic retention initiatives, given that any abrupt outflow of foreign-trained staff could destabilize the healthcare system [57]. The significant association between certain occupational factors, such as longer working hours, limited professional advancement, fewer years of experience, and elevated turnover intentions, further clarifies the relationship between workload demands and staff attrition. Although longer weekly hours predicted higher turnover intention at the bivariate level, this association dissipated after multivariable adjustment; extended shifts (e.g., 12 h rotations) exacerbate physical and emotional fatigue, potentially negating the positive effects of otherwise favorable QOL elements [58, 59]. From a policy perspective, these results underline the necessity of adopting flexible shift schedules and implementing supportive leadership practices to mitigate nurse burnout [60].

Leadership style plays a pivotal role in shaping nurses' experiences and intentions to remain in their roles [61]. Transformational leadership, characterized by supportive communication, vision-sharing, and recognition, has been shown to foster organizational commitment, reduce emotional exhaustion, and enhance job satisfaction, all of which are critical to lowering turnover intentions [62]. In contrast, autocratic or transactional leadership approaches that lack emotional intelligence may exacerbate stress and disengagement, particularly in high-pressure environments such as hospitals with long-shift demands [63, 64]. Nurse managers who adopt a transformational or servant leadership model can create psychologically safe and empowering work environments, helping nurses feel valued, heard, and motivated to stay. Investing in leadership development programs that train nurse leaders in supportive and inclusive management practices may thus be a powerful retention strategy, especially for younger staff or those in the early stages of their career who require mentoring and guidance [65].

### 5.1. Implications for Practice and Policy

#### 5.1.1. Tailored Retention Strategies

Hospital management might consider targeted interventions that address the unique challenges faced by younger, single, or less experienced nurses. Mentorship programs could ease transitions into demanding roles, while professional development tracks may enable career growth, both of which are crucial for fostering long-term commitment [66].

#### 5.1.2. Supporting Work-Life Balance

Given that extended hours were correlated with higher turnover intentions, implementing balanced shift schedules or offering part-time options may alleviate job-related stress. Such measures could be particularly effective for nurses juggling family obligations or advanced academic pursuits [67].

#### 5.1.3. Financial Incentives and Career Pathways

The positive link between higher salary and better QOL underscores the value of economic stability for nurse well-being, but salary alone did not directly reduce turnover intention in the final model. Hospitals and policymakers should develop clearly defined career pathways and competitive compensation structures to retain skilled practitioners, whether Saudi nationals or expatriates [68].

#### 5.1.4. Cultural Integration for Expatriate Nurses

Recognizing the multicultural workforce in Saudi healthcare, administrators should strengthen orientation, language support, and social integration programs that help expatriate nurses adapt more comfortably to local customs and reduce the strain on their QOL [69].

Beyond the Saudi context, healthcare systems globally should prioritize leadership development programs, flexible work arrangements, and mental health support services to enhance nurse retention across diverse settings. These strategies are critical for sustaining workforce stability in both public and private healthcare sectors.

### 5.2. Study Strengths and Future Research

This study exhibits several notable strengths. By employing validated instruments, the ProQOL and the TIS-6, within a context that is underrepresented in nursing turnover research, our investigation provides robust insights into the interplay between QOL and turnover intentions among nurses in Saudi Arabia, and the simultaneous multiple linear regression enhances causal inference by isolating independent predictors. The rigorous methodology and comprehensive data analysis strengthen the study's internal validity. Furthermore, the focus on a diverse sample from a security force hospital enhances the contextual relevance of our findings. For future research, longitudinal studies are recommended to track changes in QOL and turnover intentions over time, thereby capturing the dynamic nature of these constructs. In addition, mixed-methods approaches could enrich our understanding by exploring the nuanced experiences that underpin quantitative trends. Replicating this study across multiple healthcare settings and regions within Saudi Arabia would also enhance the generalizability of the findings and inform targeted interventions. In addition, mixed-methods approaches could enrich our understanding by exploring the nuanced experiences that underpin quantitative trends.

### 5.3. Limitations

This study has several limitations that warrant consideration. First, the research was conducted at a single security force hospital in Makkah, which may limit the generalizability of the findings to other healthcare facilities and regions within Saudi Arabia. In addition, the cross-sectional nature limits causal interpretation despite the predictive modeling approach. Second, the unique work environment characterized by long, 12-h shifts may have influenced the perceptions of QOL and turnover intentions, potentially biasing responses. Finally, the use of convenience sampling, while practical, may not fully capture the diverse experiences of nurses across different healthcare sectors, such as private hospitals, the Ministry of Health, or primary healthcare settings. Moreover, the adoption of nonprobability convenience sampling may have introduced selection bias. Nurses who were more readily available or willing to participate might have been overrepresented, potentially skewing the study's findings. This self-selection bias limits the representativeness of the sample and, consequently, the generalizability of the results.

Future research using probability-based sampling methods is recommended to mitigate these biases and provide a more comprehensive view of nurse turnover intentions across various settings. In addition, all data were collected using self-reported questionnaires, which may be subject to response bias. Participants might have provided socially desirable answers or misreported their experiences because of fear of judgment, perceived repercussions, or personal interpretation of the questions. This potential bias could affect the accuracy of the reported QOL and turnover intentions. Future studies may benefit from incorporating triangulated data sources, such as interviews or supervisor evaluations, to validate and enrich self-reported findings. In addition, cultural factors such as language barriers, social isolation, and differing expectations of workplace hierarchy may have influenced expatriate nurses' responses, potentially affecting their perceptions of QOL and turnover intentions.

### 5.4. Recommendations

Based on our findings, several recommendations are offered for healthcare policymakers and administrators. First, strategies to improve nurses' QOL should be prioritized through interventions that address workload, enhance support systems, and ensure competitive compensation. Initiatives such as flexible shift scheduling, robust mentorship programs for younger staff, and clearly defined career progression paths may reduce turnover intentions. Second, targeted efforts to integrate and support Saudi and expatriate nurses, foster inclusive work environments, and provide opportunities for professional growth are essential. Finally, future research should replicate and extend this study across multiple settings and employ longitudinal designs to inform better workforce strategies that promote sustainability and high-quality patient care.

## 6. Conclusion

The pervasive issue of nurse turnover poses critical challenges for healthcare systems worldwide, and this study underscores its significance within the Saudi Arabian context. Multivariable analysis confirmed QOL as the primary determinant of turnover intention, with younger age, single marital status, and Saudi nationality also being significant; income level and work hours were not independent predictors once other factors were controlled. Notably, younger single nurses and those experiencing lower wages and extended working hours demonstrated higher turnover intentions. These insights emphasize the importance of addressing QOL comprehensively to mitigate turnover risks and enhance workforce stability. Targeted strategies, including improved compensation, flexible scheduling, and professional development initiatives, are essential for retaining nursing talent and ensuring high-quality patient care. Implementing these retention strategies directly supports the goals of Saudi Vision 2030 by strengthening the national healthcare workforce, improving service quality, and advancing sustainable health sector development.

## Figures and Tables

**Table 1:** Sociodemographic profile of participants (*n* = 300).

Variables	*N*	%
Gender		
Female	250	83.3
Male	50	16.7
Age		
Less than 25 years	4	1.3
25–35 years	195	65.0
36 years and over	101	33.7
Marital status		
Single	77	25.7
Married	212	70.7
Divorced or widowed	11	3.7
Nationality		
Saudi	63	21.0
Non-Saudi	237	79.0
Please indicate how many dependent children you have		
None	125	41.7
1–3	162	54.0
More than 3	13	4.3
Highest level of qualification		
Associate degree	40	13.3
Bachelor's degree	230	76.7
Postgraduate degrees	30	10.0
Salary range per month		
SR 2500–5000 (600–1333 US$)	63	21.0
SR 5001–10,000 (1334–2666 US$)	163	54.3
SR 10,001–15,000 (2667–4000 US$)	58	19.3
> SR 15,001 (4000 US$)	16	5.3
How many hours do you work in a week?		
Less than 50 h	216	72.0
50 h and more	84	28.0
Current role		
Staff nurse	265	88.3
Head nurse or charge nurse	20	6.7
Nurse manager (supervisor, deputy, and chief nursing)	3	1.0
Others, please indicate (nursing education, infection control nurse, and quality control nurses)	12	4.0
Number of years of experience		
Less than 1 year	11	3.7
1– > 5 years	72	24.0
5–10 years	131	43.7
More than 10 years	86	28.7

Abbreviations: QOL = quality of life, SR = Saudi riyal, TIS = turnover intention scale.

**Table 2:** Multiple linear regression predicting turnover-intention scores among registered nurses (*n* = 300).

Predictor (entered simultaneously)	*B*	SE *B*	*β*	95% CI for *B*	*t*	*p* value
Intercept	9.84	1.70	—	6.50–13.18	5.79	*p* ≤ 0.001
Quality of life (ProQOL total)	0.17	0.02	0.42	0.13–0.21	7.98	*p* ≤ 0.001^∗^
Age (years)	−0.08	0.03	−0.19	−0.13–0.03	−3.15	0.0012^∗^
Marital status^†^ (single = 1)	1.27	0.49	0.15	0.31–2.23	2.58	0.010^∗^
Nationality^‡^ (Saudi = 1)	1.05	0.44	0.13	0.18–1.92	2.37	0.018^∗^
Monthly income^§^ (SR thousands)	−0.04	0.09	−0.03	−0.22–0.14	−0.46	0.645
Years of experience	−0.02	0.04	−0.02	−0.10–0.06	−0.49	0.623

*Note:* Positive *B* values indicate higher TIS scores (greater turnover intention) with increasing predictor values; negative *B* values indicate the reverse. Model summary: *R* = 0.62; *R*^2^ = 0.39; adjusted *R*^2^ = 0.38; *F* (6, 293) = 31.4, *p* ≤ 0.001.

^∗^Significant at *α* = 0.05.

^†^Reference category = married/divorced/widowed.

^‡^Reference category = non-Saudi.

^§^Income entered as a continuous variable (mid-point of self-reported salary range divided by 1000).

**Table 3:** Relationship between nurses' sociodemographic characteristics, turnover intention scale (TIS), and quality of life (QOL) scores.

		TIS		QOL	
		Mean ± SD	*p* value	Mean ± SD	*p* value
Gender	Female	17.77 ± 5.42	0.82	90.11 ± 10.48	0.11
	Male	17.58 ± 4.56		92.68 ± 9.84	

Age	Less than 25 years	21.75 ± 3.95	0.001^∗^	97.25 ± 5.32	0.42
	25–35 years	18.47 ± 4.94		90.56 ± 10.63	
	36 years and over	16.17 ± 5.61		90.23 ± 10.09	

Marital status	Single	19.62 ± 4.35	0.001^∗^	93.34 ± 9.65	0.01^∗^
	Married	17.15 ± 5.46		89.42 ± 10.68	
	Divorced or widowed	15.82 ± 4.51		92.45 ± 5.50	

Nationality	Saudi	20.16 ± 5.01	0.001^∗^	94.06 ± 10.84	0.001^∗^
	Non-Saudi	17.09 ± 5.17		89.60 ± 10.10	

Dependent children	None	18.69 ± 5.08	0.02^∗^	91.22 ± 10.34	0.26
	1–3	17.19 ± 5.22		89.75 ± 10.42	
	More than 3	15.46 ± 6.57		93.77 ± 10.56	

Highest level of qualification	Associate degree	15.15 ± 5.41	0.001^∗^	87.53 ± 12.09	0.14
	Bachelor's degree	18.27 ± 5.17		90.96 ± 10.09	
	Postgraduate degrees	17.13 ± 5.03		91.33 ± 10.05	

Salary range per month	SR 2500–5000	18.94 ± 4.48	0.01^∗^	89.89 ± 9.95	0.049^∗^
	SR 5001–10,000	17.09 ± 5.45		89.48 ± 10.53	
	SR 10,001–15,000	18.86 ± 5.12		93.78 ± 9.88	
	>SR 15,001	15.50 ± 5.57		92.13 ± 11.09	

Hours worked in a week	Less than 50 h	17.27 ± 5.31	0.01^∗^	90.05 ± 9.70	0.19
	50 h and more	18.93 ± 5.03		91.81 ± 12.00	

Current role	Staff nurse	17.72 ± 5.38	0.20	90.43 ± 10.44	0.92
	Head nurse or charge nurse	18.30 ± 4.54		91.85 ± 10.98	
	Nurse manager	11.67 ± 2.52		92.67 ± 11.06	
	Other	18.75 ± 3.70		90.17 ± 9.48	

Number of years of experience	Less than 1 year	18.00 ± 5.76	0.001^∗^	93.55 ± 10.71	0.29
	1– > 5 years	19.61 ± 4.53		91.83 ± 9.46	
	5–10 years	17.53 ± 5.38		89.38 ± 11.51	
	More than 10 years	16.45 ± 5.29		90.84 ± 9.22	

Abbreviations: QOL = quality of life, SD = standard deviation, TIS = turnover intention scale.

^∗^ *p* value < 0.05.

## Data Availability

The datasets generated and analyzed during the current study are available from the corresponding author upon reasonable request.

## Supporting Information

Supporting File 1: Full three-part questionnaire used for data collection (Part I: Demographic and Professional Characteristics; Part II: Professional Quality of Life Scale V5; and Part III: Turnover Intention Scale-6 (TIS-6)).

## References

[1] Nemati-Vakilabad R., Kamalifar E., Jamshidinia M., Mirzaei A. (2025). Assessing the Relationship Between Nursing Process Competency and Work Environment Among Clinical Nurses: a Cross-Sectional Correlational Study. *BMC Nursing*.

[2] Bellegarde-Armstrong K., Potter T., Professor C. (2025). Nurses as UN Champions: Increasing Nursing Presence and Influence at the United Nations. *International Nursing Review*.

[3] O’Brien-Pallas L., Griffin P., Shamian J. (2006). The Impact of Nurse Turnover on Patient, Nurse, and System Outcomes: a Pilot Study and Focus for a Multicenter International Study. *Policy, Politics, & Nursing Practice*.

[4] Peng X., Ye Y., Ding X., Chandrasekaran A. (2023). The Impact of Nurse Staffing on Turnover and Quality: an Empirical Examination of Nursing Care Within Hospital Units. *Journal of Operations Management*.

[5] Albalawi A. M., Pascua G. P., Alsaleh S. A. (2024). Factors Influencing Nurses Turnover in Saudi Arabia: a Systematic Review. *Nursing Forum*.

[6] Elsharkawy N. B., Alruwaili A. N., Elsayed Ramadan O. M., Alruwaili M. M., Alhaiti A., Abdelaziz E. M. (2025). Barriers to Reporting Workplace Violence: a Qualitative Study of Nurses’ Perceptions in Tertiary Care Settings. *BMC Nursing*.

[7] Ki Y., Mcaleavey A. A., Øien J. M. T., Moger T. A., Moltu C. (2025). Measuring Health-Related Quality of Life: a Qualitative Study of Mental Health Patients’ Experiences of Impacts of Mental Health Issues. *International Journal of Qualitative Studies on Health and Well-Being*.

[8] Alsadaan N., Mohamed O., Ramadan E. (2025). Barriers and Facilitators in Implementing Evidence-Based Practice: a Parallel Cross-Sectional Mixed Methods Study Among Nursing Administrators. *BMC Nursing*.

[9] Rodrigues M. C. J., Rocha A. C. R., Couto C. R. (2025). Measuring Health-Related Quality of Life Among University Students: a Scoping Review Protocol. *Systematic Reviews*.

[10] Memisevic H., Ibralic I. (2025). Quality of Life and Disability. *The Palgrave Encyclopedia of Disability*.

[11] Alhindi A. A., Mahmud I., Altakroni H. (2024). Assessment of Nursing Shortage and Calculation Methods in Saudi Arabia: a Cross-Sectional Study in Government Hospitals. *Cureus*.

[12] Alblihed M., Alzghaibi H. A. (2022). The Impact of Job Stress, Role Ambiguity and Work–Life Imbalance on Turnover Intention During COVID-19: a Case Study of Frontline Health Workers in Saudi Arabia. *International Journal of Environmental Research and Public Health*.

[13] Alshehry A. S., Id A. (2024). Association of Personal and Professional Factors, Resilience and Quality of Life of Registered Nurses in a University Medical City in the Kingdom of Saudi Arabia. *PLoS One*.

[14] Bujang M. A., Lai W. H., Tiong X. T. (2025). Quality of Life and Overall Well-Being Between Healthy Individuals and Patients with Varied Clinical Diagnoses. *BMC Public Health*.

[15] Bujang M. A., Husin M. (2024). Health-Related Quality of Life with Six Domains (HRQ-6D): Features and Applications. *Handbook of the Behavior and Psychology of Disease*.

[16] Jachmann A., Loser A., Mettler A., Exadaktylos A., Müller M., Klingberg K. (2025). Burnout, Depression, and Stress in Emergency Department Nurses and Physicians and the Impact on Private and Work Life: a Systematic Review. *JACEP Open*.

[17] Zhang Y., Fu Y., Zheng X., Shi X., Liu J., Chen C. (2025). The Impact of Nursing Work Environment, Emotional Intelligence, and Empathy Fatigue on Nurses’ Presenteeism: a Structural Equation Model. *BMC Nursing*.

[18] Alzahrani A. A., Pavlova M., Alsubahi N., Ahmad A., Groot W. (2025). Impact of the Cooperative Health Insurance System in Saudi Arabia on Universal Health Coverage—A Systematic Literature Review. *Health Care*.

[19] Laviña S. M. S. (2024). Measuring Quality of Life Through Validated Tools. *Acta Medica Philippina*.

[20] Lazzari M., Alvarez J. M., Ruggieri S. (2022). Predicting and Explaining Employee Turnover Intention. *International Journal of Data Science and Analytics*.

[21] Mafula D., Arifin H., Chen R. (2025). Prevalence and Moderating Factors of Turnover Rate and Turnover Intention Among Nurses Worldwide: a Meta-Analysis. *Journal of Nursing Regulation*.

[22] AbdELhay E. S., Taha S. M., El-Sayed M. M., Helaly S. H., AbdELhay I. S. (2025). Nurses Retention: the Impact of Transformational Leadership, Career Growth, Work Well-Being, and Work-Life Balance. *BMC Nursing*.

[23] Al-Mansour K. (2021). Stress and Turnover Intention Among Healthcare Workers in Saudi Arabia During the Time of COVID-19: Can Social Support Play a Role?. *PLoS One*.

[24] Al-Suraihi W. A., Samikon S. A., Al-Suraihi A.-H. A., Ibrahim I. (2021). Employee Turnover: Causes, Importance and Retention Strategies. *European Journal of Business and Management Research*.

[25] Alnehabi M., Al-Mekhlafi A.-B. A. (2025). Exploring the Influence of the Critical Factors on Intentions of Employee Turnover in the Banking Segment: a Comprehensive Mediating Analysis. *BMC Psychol*.

[26] Sainju B., Hartwell C., Edwards J. (2021). Job Satisfaction and Employee Turnover Determinants in Fortune 50 Companies: Insights from Employee Reviews from Indeed.Com. *Decision Support Systems*.

[27] Alluhidan M., Tashkandi N., Alblowi F. (2020). Challenges and Policy Opportunities in Nursing in Saudi Arabia. *Human Resources for Health*.

[28] Jeilani A., Hussein A. (2025). Impact of Digital Health Technologies Adoption on Healthcare Workers’ Performance and Workload: Perspective with DOI and TOE Models. *BMC Health Services Research*.

[29] Pressley C., Garside J. (2023). Safeguarding the Retention of Nurses: a Systematic Review on Determinants of Nurse’s Intentions to Stay. *Nursing Open*.

[30] Poku C. A., Alem J. N., Poku R. O., Osei S. A., Amoah E. O., Ofei A. M. A. (2022). Quality of Work-Life and Turnover Intentions Among the Ghanaian Nursing Workforce: a Multicentre Study. *PLoS One*.

[31] Ibrahim Alzamel L. G., Abdullah K. L., Chong M. C., Chua Y. P. (2020). The Quality of Work Life and Turnover Intentions Among Malaysian Nurses: the Mediating Role of Organizational Commitment. *Journal of the Egyptian Public Health Association*.

[32] Specchia M. L., Cozzolino M. R., Carini E. (2021). Leadership Styles and Nurses’ Job Satisfaction. Results of a Systematic Review. *International Journal of Environmental Research and Public Health*.

[33] Gottlieb L. N., Gottlieb B., Bitzas V. (2021). Creating Empowering Conditions for Nurses with Workplace Autonomy and Agency: How Healthcare Leaders Could Be Guided by Strengths-Based Nursing and Healthcare Leadership (SBNH-L). *Journal of Healthcare Leadership*.

[34] Elfios E., Asale I., Merkine M. (2024). Turnover Intention and Its Associated Factors Among Nurses in Ethiopia: a Systematic Review and Meta-Analysis. *BMC Health Services Research*.

[35] Lee C., Lee B., Choi I., Kim J. (2023). Exploring Determinants of Job Satisfaction: a Comparison Between Survey and Review Data. *Sage Open*.

[36] Midgette A. J. (2020). Chinese and South Korean Families’ Conceptualizations of a Fair Household Labor Distribution. *Journal of Marriage and Family*.

[37] Smith P. B., Bond M. H., Shengtao Wu M., Bevington Smith P., Harris Bond M. (2019). Cultures and Persons: Characterizing National and Other Types of Cultural Difference Can Also Aid Our Understanding and Prediction of Individual Variability. *Frontiers in Psychology*.

[38] Mazibu A., Downing C., Rasesemola R. (2024). Expatriate Professional Nurses’ Experiences of Preceptorship in a Tertiary Hospital in Saudi Arabia. *Saudi Journal for Health Sciences*.

[39] Al-Dossary R. N. (2022). The Relationship Between Nurses’ Quality of Work-Life on Organizational Loyalty and Job Performance in Saudi Arabian Hospitals: a Cross-Sectional Study. *Frontiers in Public Health*.

[40] Almansour H., Aldossary A., Holmes S., Alderaan T. (2023). Migration of Nurses and Doctors: Pull Factors to Work in Saudi Arabia. *Human Resources for Health*.

[41] Demerouti E., Bakker A. B., Nachreiner F., Schaufeli W. B. (2001). The Job Demands-Resources Model of Burnout. *Journal of Applied Psychology*.

[42] Glicken M. D., Robinson B. C. (2013). Understanding Job Stress, Job Dissatisfaction, and Worker Burnout. *Treating Worker Dissatisfaction During Economic Change*.

[43] Gou J., Zhang X., He Y., He K., Xu J. (2024). Effects of Job Demands, Job Resources, Personal Resources on Night-Shift Alertness of ICU Shift Nurses: a Cross-sectional Survey Study Based on the Job Demands-Resources Model. *BMC Nursing*.

[44] Thompson S. K. (2012). *Sampling*.

[45] Hemsworth D., Baregheh A., Aoun S., Kazanjian A. (2018). A Critical Enquiry into the Psychometric Properties of the Professional Quality of Life Scale (ProQol-5) Instrument. *Applied Nursing Research*.

[46] Singh J., Karanika-Murray M., Baguley T., Hudson J. (2024). A Psychometric Evaluation of Professional Quality of Life Scale Version 5 (ProQOL 5) in a UK-Based Sample of Allied Mental Health Professionals. *Current Psychology*.

[47] Bothma C. F. C., Roodt G. (2013). The Validation of the Turnover Intention Scale. *SA Journal of Human Resource Management*.

[48] Scanlan J. N., Still M. (2019). Relationships Between Burnout, Turnover Intention, Job Satisfaction, Job Demands and Job Resources for Mental Health Personnel in an Australian Mental Health Service. *BMC Health Services Research*.

[49] van Kraaij J., de Vries N., Wessel H. (2025). Enhancing Work Environments and Reducing Turnover Intention: a Multicenter Longitudinal Cohort Study on Differentiated Nursing Practices in Dutch Hospitals. *BMC Nursing*.

[50] Bagdžiūnienė D., Žukauskaitė I., Bulotaitė L., Sargautytė R. (2025). Study and Personal Resources of University Students’ Academic Resilience and the Relationship with Positive Psychological Outcomes. *Frontiers in Psychology*.

[51] Alessandri G., Borgogni L., Latham G. P. (2025). Direct and Indirect Longitudinal Relationships Among Self-Efficacy, Job Performance and Career Advancements. *International Journal of Psychology: Journal International de Psychologie*.

[52] Almubark S., Booth A., Wood E. (2025). Turnover and Turnover Intention Among Nurses Working in Saudi Arabia: a Qualitative Evidence Synthesis. *Journal of Advanced Nursing*.

[53] Alreshidi N. M., Alrashidi L. M., Alanazi A. N., Alshammri E. H. (2021). Turnover Among Foreign Nurses in Saudi Arabia. *Journal of Public Health Research*.

[54] Alqarawi N., Alasqah I., Al Harbi A. S., Adolfo C. S., Almazan J. U. (2025). Examining the Relationship Between Nursing Staff Demographics, Work Characteristics, and Toxic Leadership in Saudi Arabia: A Cross-Section Approach. *BMC Nursing*.

[55] Farahani M. A., Nargesi S., Saniee N., Dolatshahi Z., Heidari Beni F., Shariatpanahi S. (2024). Factors Affecting Nurses Retention During the COVID‐19 Pandemic: A Systematic Review. *Human Resources for Health*.

[56] Deng J., Wang P., Tian X., Li K., Yang L., Ding S. (2024). Turnover Intention and Its Influencing Factors Among Male Nurses in China: A National-Scale Descriptive Study. *BMC Nursing*.

[57] Ramadan O. M. E., Alruwaili M. M., Alruwaili A. N., Elsehrawy M. G., Alanazi S. (2024). Facilitators and Barriers to AI Adoption in Nursing Practice: A Qualitative Study of Registered Nurses’ Perspectives. *BMC Nursing*.

[58] Van Der Heijden B., Brown Mahoney C., Xu Y. (2019). Impact of Job Demands and Resources on Nurses’ Burnout and Occupational Turnover Intention Towards an Age-Moderated Mediation Model for the Nursing Profession. *International Journal of Environmental Research and Public Health*.

[59] Wong K. P., Zhang B., Xie Y. J. (2024). Impacts of Job Demands on Turnover Intention Among Registered Nurses in Hong Kong Public Hospitals: Exploring the Mediating Role of Burnout and Moderating Effect of Pay Level Satisfaction. *Journal of Nursing Management*.

[60] Dall’Ora C., Ejebu O. Z., Ball J., Griffiths P. (2023). Shift Work Characteristics and Burnout Among Nurses: Cross-Sectional Survey. *Occupational Medicine*.

[61] Pattali S., Sankar J. P., Al Qahtani H., Menon N., Faizal S. (2024). Effect of Leadership Styles on Turnover Intention Among Staff Nurses in Private Hospitals: the Moderating Effect of Perceived Organizational Support. *BMC Health Services Research*.

[62] Jankelová N., Joniaková Z. (2021). Communication Skills and Transformational Leadership Style of First-Line Nurse Managers in Relation to Job Satisfaction of Nurses and Moderators of This Relationship. *Healthcare*.

[63] Alluhaybi A., Wilson A., Usher K., Durkin J. (2023). Impact of Nurse Manager Leadership Styles on Work Engagement: a Systematic Literature Review. *Journal of Nursing Management*.

[64] Alsadaan N., Ramadan O. M. E., Alqahtani M. (2024). From Incivility to Outcomes: Tracing the Effects of Nursing Incivility on Nurse Well-Being, Patient Engagement, and Health Outcomes. *BMC Nursing*.

[65] Hafsteinsdóttir T. B., Huntink E., Schoonhoven L. (2025). Leadership Mentoring in Nursing Research Influencing Leadership Development, Career Development, and Research Productivity of Phd Prepared Nurses: a Pretest–Posttest Study. *Nursing Outlook*.

[66] Bell S., Gorsuch P., Beckett C., McComas A., Boss K., Rose K. (2025). An Evidence-Based Initiative to Reduce New Graduate Nurse Turnover: Implementation of a Mentorship Program. *Worldviews on Evidence-Based Nursing*.

[67] Bae S. H. (2024). Nurse Staffing, Work Hours, Mandatory Overtime, and Turnover in Acute Care Hospitals Affect Nurse Job Satisfaction, Intent to Leave, and Burnout: a Cross-Sectional Study. *International Journal of Public Health*.

[68] Alanazi R., Bahari G., Alzahrani Z. A. (2023). Exploring the Factors Behind Nurses’ Decision to Leave Clinical Practice: Revealing Causes for Leaving and Approaches for Enhanced Retention. *Healthcare*.

[69] Eugenia Foo Bsn H., Nurse Yong Shian Goh R., Professor A., Shian Goh Y. (2024). Facilitating Acculturation of Internationally Educated Nurses: a Meta-Synthesis of Social Integration Strategies. *International Nursing Review*.

