# Supplementary material for: The Relationship Between Quality of Life and Nurses' Turnover Intentions: A Cross-Sectional Study
Source: J Nurs Manag. 2025 Aug 1;2025:4951493. doi: 10.1155/jonm/4951493 (PMC12334292; doi:10.1155/jonm/4951493)
Supplement: Supporting Information — Additional supporting information can be found online in the Supporting Information section. [file 4951493.f1.pdf]

Comprehensive Questionnaire

Below is the complete questionnaire used in the study "The Relationship Between Quality of Life and Nurse Turnover Intentions: A Study at a Makkah Security Force Hospital." This questionnaire comprises three sections (I) Demographic and Professional Characteristics, (II) Professional Quality of Life Scale (ProQOL, Version 5), and (III) Turnover Intention Scale (TIS-6). Each section was designed to capture relevant data in a rigorous and methodologically sound manner. All participants were informed of the study's aims and provided written consent prior to completing the questionnaire.

Part I: Demographic and Professional Characteristics

1. **Gender**
    - Male
    - Female
2. **Age (years)**
    - Under 25
    - 25–35
    - 36 and over
3. **Marital Status**
    - Single (never married)
    - Married
    - Divorced/Widowed
4. **Number of Dependent Children**
    - None
    - 1–3
    - More than 3
5. **Nationality**
    - Saudi
    - Non-Saudi (Please specify nationality if comfortable)
6. **Highest Educational Qualification**
    - Diploma/Associate degree in Nursing
    - Bachelor's degree in Nursing (BSN)
    - Post-graduate degree in Nursing (Master's or Doctorate)
    - Other (please specify)
7. **Current Role/Position**
    - Staff Nurse
    - Head Nurse or Charge Nurse
    - Nurse Manager (e.g., Supervisor, Deputy, Chief Nursing Officer)
    - Other (please specify)
8. **Number of Years of Experience in Nursing**
    - Less than 1 year
    - 1–5 years
    - 5–10 years
    - More than 10 years
9. **Weekly Working Hours**
    - Less than 50 hours per week
    - 50 hours or more per week

10. **Monthly Salary Range (in Saudi Riyals)**

- 2,500–5,000 (approx. 600–1,333 USD)
- 5,001–10,000 (approx. 1,334–2,666 USD)
- 10,001–15,000 (approx. 2,667–4,000 USD)
- Above 15,000 (above 4,000 USD)

11. **Self-Reported Nursing Knowledge/Competence**

- Beginner
- Intermediate
- Advanced

Part II: Professional Quality of Life Scale (ProQOL) – Version 5

The following 30 items measure aspects of your professional quality of life (ProQOL). Please read each statement carefully and indicate how frequently you have experienced these feelings in the **past 30 days**, using the scale below:

1 = **Never**
2 = **Rarely**
3 = **Sometimes**
4 = **Often**
5 = **Very Often**

Please circle or select one response per item.

1. I am happy.
2. I am preoccupied with more than one person I help.
3. I get satisfaction from being able to help people.
4. I feel connected to others.
5. I jump or am startled by unexpected sounds.
6. I feel invigorated after working with those I help.
7. I find it difficult to separate my personal life from my life as a helper.
8. I am not as productive at work because I am losing sleep over traumatic experiences of a person I help.
9. I think that I might have been affected by the traumatic stress of those I help.
10. I feel trapped by my job as a helper.
11. Because of my helping, I have felt “on edge” about various things.
12. I like my work as a helper.
13. I feel depressed because of the traumatic experiences of the people I help.
14. I feel as though I am experiencing the trauma of someone I have helped.
15. I have beliefs that sustain me.
16. I am pleased with how I am able to keep up with helping techniques and protocols.
17. I am the person I always wanted to be.
18. My work makes me feel satisfied.
19. I feel worn out because of my work as a helper.
20. I have happy thoughts and feelings about those I help and how I could help them.
21. I feel overwhelmed because my case/workload seems endless.
22. I believe I can make a difference through my work.
23. I avoid certain activities or situations because they remind me of frightening experiences of the people I help.
24. I am proud of what I can do to help.
25. As a result of my helping, I have intrusive, frightening thoughts.
26. I feel “bogged down” by the system.
27. I have thoughts that I am a “success” as a helper.
28. I can’t recall important parts of my work with trauma victims.
29. I am a very caring person.
30. I am happy that I chose to do this work.

Scoring and Dimensions

- **Compassion Satisfaction**: Items 3, 6, 12, 16, 18, 20, 22, 24, 27, 29
- **Burnout**: Items 1, 8, 10, 12, 17, 19, 21, 26, 29 (reverse-coded as needed)

- **Secondary Traumatic Stress**: Items 2, 5, 7, 9, 11, 13, 14, 23, 25, 28
- **Moral Distress**: Some versions treat items relating to moral conflict or distress differently; please see Stamm (2010) for details on dimension assignment.
- **Perceived Support**: Items vary; consult the ProQOL manual for item distribution.

For details on calculating subscale scores, refer to **Stamm (2010)** or the ProQOL manual.

Part III: Turnover Intention Scale (TIS-6)

Below are six statements concerning your current job. Please indicate the extent to which each statement applies to you, using the following 5-point Likert scale:

1 = **Strongly Disagree**
2 = **Disagree**
3 = **Neutral**
4 = **Agree**
5 = **Strongly Agree**

1. I often think about quitting my job.
2. I plan to leave my current job soon.
3. I have a desire to quit my job.
4. It would be easy for me to leave my current job if I wanted to.
5. I am actively searching for an alternative to my current job.
6. I expect to remain in my current job for a long time. (reverse-coded)

Scoring

- Reverse-code item 6.
- Sum all six responses to form a total Turnover Intention score (range = 6–30).
- Higher scores indicate stronger turnover intentions.

Instructions for Completing the Questionnaire

1. **Confidentiality**: Your responses will be treated with strict confidentiality. No identifying information (such as names or contact details) will be linked to your answers.
2. **Voluntary Participation**: Participation is entirely voluntary. You are free to skip any question you are not comfortable answering or withdraw at any time before submitting the questionnaire.
3. **Duration**: Completing all three sections should take approximately 10–15 minutes.
4. **Submission**: Please return or submit the completed questionnaire according to the instructions provided (e.g., online form submission or sealed envelope).

End of Questionnaire

Please note that the ProQOL and TIS-6 instruments are used herein with acknowledgment of their respective authors. For comprehensive scoring details and factor interpretations, refer to the standard manuals (Stamm, 2010; Bothma & Roodt, 2013). This supplementary file serves as a complete reference for researchers and institutional review boards to verify data collection procedures.
